# Low prevalence of high blood pressure in pregnant women in Burkina Faso: a cross-sectional study

**DOI:** 10.1186/s12884-022-05242-5

**Published:** 2022-12-21

**Authors:** Franck Garanet, Sekou Samadoulougou, Adama Baguiya, Bruno Bonnechère, Tieba Millogo, Jean-Marie Degryse, Fati Kirakoya-Samadoulougou, Seni Kouanda

**Affiliations:** 1 Centre National de la Recherche Scientifique et Technologique (CNRST), Institut de Recherche en Sciences de la Santé (IRSS), Département Biomédical et Santé Publique, Ouagadougou, Burkina Faso; 2 Centre de Recherche en Epidémiologie, Biostatistiques et Recherche Clinique, Ecole de Santé Publique, Université libre de Bruxelles, Bruxelles, Belgique; 3 Université Ouaga1 Joseph Ki-Zerbo, Ecole Doctorale Science de la Santé (ED2S), Laboratoire de Santé Publique (LASAP), Ouagadougou, Burkina Faso; 4 Centre for Research on Planning and Development (CRAD), Laval University, Quebec, G1V 0A6 Canada; 5 Evaluation Platform On Obesity Prevention, Quebec Heart and Lung Institute, Quebec, G1V 4G5 Canada; 6 Institut Africain de Santé Publique (IASP), Ouagadougou, Burkina Faso; 7 REVAL Rehabilitation Research Center, Faculty of Rehabilitation Sciences, Hasselt University, Diepenbeek, Belgium; 8 Institut de Recherche Sciences et Société (IRSS), Université Catholique de Louvain, Bruxelles, Belgique; 9 Department of Public Health and Primary Care - Katholieke Universiteit Leuven, Leuven, Belgique

**Keywords:** High blood pressure, Pregnancy, Prevalence, Rural and Semi-urban, Burkina Faso

## Abstract

**Background:**

High blood pressure (HBP) during pregnancy causes maternal and fetal mortality. Studies regarding its prevalence and associated factors in frontline level health care settings are scarce. We thus aimed to evaluate the prevalence of HBP and its associated factors among pregnant women at the first level of the health care system in Burkina Faso.

**Methods:**

This cross-sectional study was conducted in six health facilities between December 2018 and March 2019. HBP was defined as systolic blood pressure ≥ 140 mmHg and/or diastolic blood pressure ≥ 90 mmHg. Multivariable logistic regression analysis was performed to identify factors associated with HBP.

**Results:**

A total of 1027 pregnant women were included. The overall prevalence of HBP was 1.4% (14/1027; 95% confidence interval [CI] 0.7–2.3), with 1.6% (7/590; 95% CI 0.8–3.3) in rural and 1.2% (7/437; 95% CI 0.6- 2.5) in semi-urban areas. The prevalence was 0.7% (3/440; 95% CI 0.2–2.1) among women in the first, 1.5% (7/452; 95% CI 0.7–3.2) in the second and 3% (4/135; 95% CI 1.1–7.7) in the third trimester. In the multivariable analysis, pregnancy trimester, maternal age, household income, occupation, parity, and residential area were not associated with HBP during pregnancy.

**Conclusion:**

The prevalence of HBP among pregnant women at the first level of health system care is significantly lower compared to prevalence’s from hospital studies. Public health surveillance, primary prevention activities, early screening, and treatment of HDP should be reinforced in all health facilities to reduce the burden of adverse pregnancy outcomes in Burkina Faso.

## Background

Maternal mortality is unacceptably high. Worldwide, approximately 295,000 women died during pregnancy, childbirth and the postpartum period in 2017 [1]. Every day, approximately 810 women die from preventable causes related to pregnancy and childbirth [1].

The vast majority of these deaths (94%) occur in low-resource settings and most could have been prevented [1]. Numerous factors, such as hypertensive disorders of pregnancy (HDP), have been identified as being responsible for this mortality [2–5]. According to a recent report from the World Health Organization (WHO), HDP is the leading cause of maternal mortality in some African settings [3]. HDP is a multisystem disorder that includes gestational hypertension, chronic hypertension with superimposed pre-eclampsia and pre-eclampsia. HDP contribute to 14% of maternal mortality worldwide and 16% in sub-Saharan Africa [3, 4]. Globally, 2.7% of pregnant women suffer from HDP, whereas the incidence of chronic hypertension is 0.29%; the prevalence of HDP is 2.16% in pre-eclampsia and 0.28% in eclampsia [6]. Many studies have investigated the prevalence of high blood pressure (HBP) in pregnant women, with most studies being conducted in tertiary hospitals [7–9]. Very different prevalence of HDP has been reported, ranging from 1.3% in China to 26.7% in Tanzania [10, 11]; in 2022, a prevalence of 0.3% in women of childbearing age was reported in sub-Saharan Africa [12]. Few studies, however, have investigated HBP prevalence at the healthcare system level; furthermore, no studies have been conducted in Burkina Faso.

The primary goal of WHO recommendations is to improve the quality of care and outcomes for pregnant women, particularly related with the management of HDP [13, 14]. Women with HDP should have a comprehensive care plan that includes prenatal counselling, frequent visits during pregnancy, timely birth, appropriate intrapartum monitoring and care and postpartum follow-up. Management involves advice during each stage of pregnancy to ensure that the woman is aware of the risks to herself and her fetus [13, 15]. In an era where data govern health priorities, evaluation of the prevalence of HBP in particular settings has important implications for surveillance [16, 17]. This study was conducted to determine the prevalence of HBP among pregnant women at the first level of the healthcare system in Burkina Faso and to investigate associated risk factors.

## Methods

### Study design and population

**Fig. 1:**
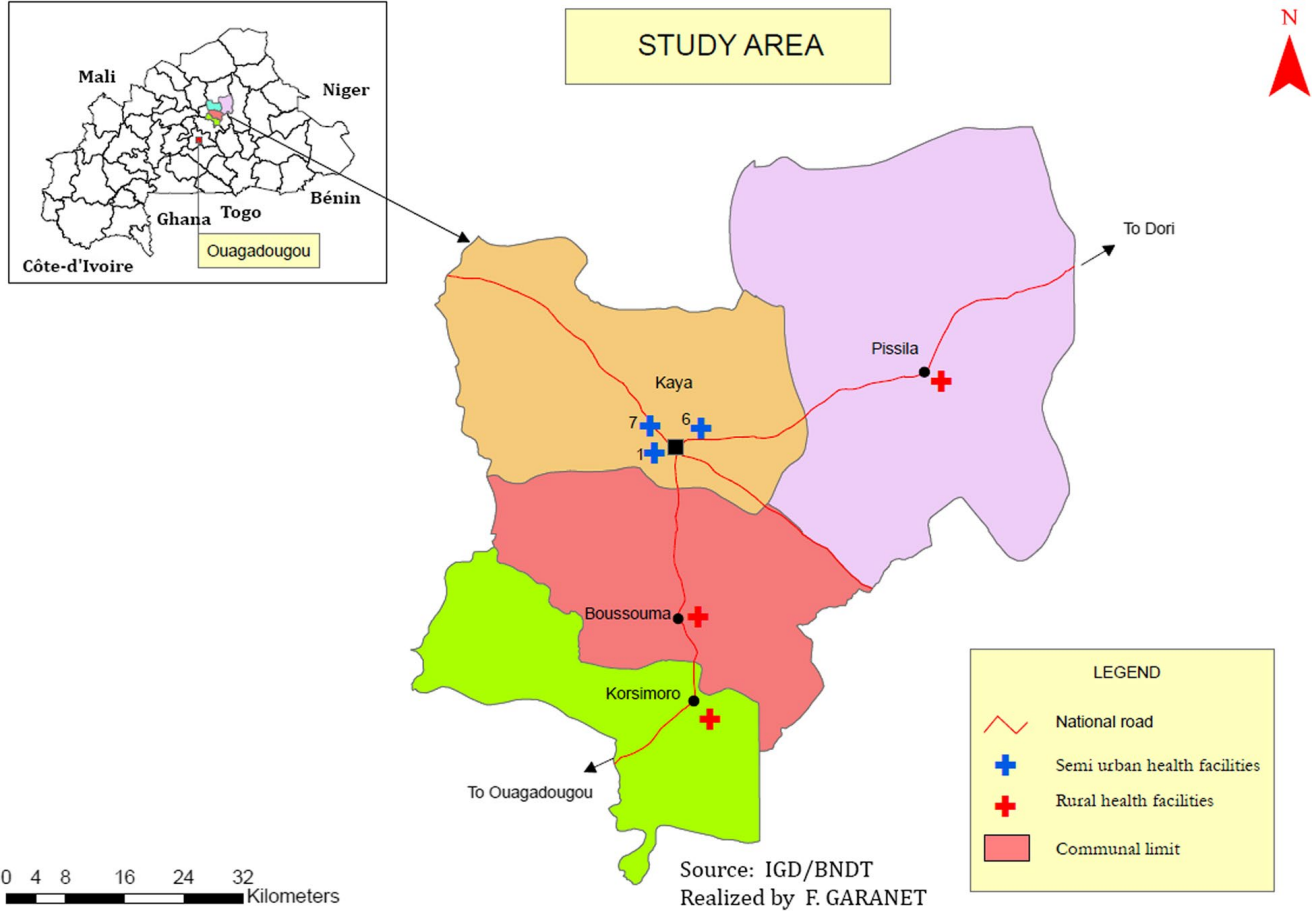
Study area

This cross-sectional study was conducted between December 2018 and March 2019. The study population comprised pregnant women attending antenatal care (ANC) in the three largest semi-urban health facilities of Kaya town (the medical centers of sectors 1, 6 and 7) and the three largest rural health facilities around Kaya town (Boussouma, Pissila and Korsimoro) (Fig. 1).

### Eligibility criteria

Eligible participants were recruited consecutively in each health facility. All women receiving ANC, regardless of the stage of pregnancy, were included after providing informed consent.

### Calculation of sample size

There are wide ranges in the prevalence of HBP during pregnancy and we did not deem any of these as being representative for our study setting [4]. We considered the prevalence of 9.6% of HBP in the teaching hospital of Yalgado, Ouagadougou, Burkina Faso and a non-response rate of 10% with a type one error set at 5% with graph effect [18]. The minimum sample size was 880 participants.

### Data collection

The women were invited to face-to-face interviews after identification and signing of consent. Clinical data regarding ANC were retrieved from women’s health booklets and from the antenatal clinic's register in the health facilities. A structured questionnaire was used to collect data. Six health workers with experience in data collection and speaking a local language conducted face-to-face interviews to obtain demographic characteristics; also, blood pressure was measured at the end of the interview, after approximately 15 min of rest.

An electronic blood pressure monitor (Omron M3 Intellisense device) with an adult-sized cuff was used to measure blood pressure with the participant in a sitting position. The initial blood pressure was measured in each arm to determine the arm with the highest blood pressure. Two other measurements were successively performed on the retained arm with an interval of one minute between each measurement. Average of the last two measurements was considered as the participant's blood pressure.

### Data quality assurance

All health workers were trained by the research team to use the tools for various data collection techniques. A pre-test was conducted in a health center that did not participate in the study. A manual containing all interview and measurement procedures was given to each interviewer.

### Statistical analysis

HBP was defined as systolic blood pressure ≥ 140 mmHg and/or diastolic blood pressure ≥ 90 mmHg [19]. The score of a household’s socioeconomic status was computed based on 20 items using principal component analysis. These 20 items included the presence of a bucket, bowls, cup, gas fireplace, bed, mattress, table, chair, functional radio, functional clock, functional lamp, functional television, functional bicycle, functional motorcycle, functional telephone, functional cart, functional wheelbarrow, donkey, horse, poultry, sheep and goats.

The variables used for this study were recorded as follows: age (< 19 years, 20–29 years and ≥ 30 years), age at first pregnancy (13–19 years and 20–36 years), education level (none, primary and secondary, higher), level of wealth (poor, medium, rich), occupation (employed and not employed), pregnancy trimester (first trimester, second trimester and third trimester), parity (one or more than one) and place of residence (rural and semi-urban). We used a multivariable logistic regression model (maternal age, household income, occupation, pregnancy trimester, parity and residential area) to determine the factors associated with the presence of HBP. All statistical analyses were performed using Stata V.14.1 (Stata 2014: Revision 01 dec 2015. 147 College Station, Texas, USA) [20].

## Results

Among the six health facilities, 1027 women received antenatal care and all agreed to participate. Mean age of the participants was 25.8 ± 6.0 years and of them, 14.4% (148/1027) were < 20 years, out of whom 17.4% (76/437) lived in semi-urban areas, and 12.2% (72/590) in rural areas. Most participants were younger than 20 years in the first pregnancy 71.8% (732/1027) and had not received formal education (63.3%; 650/1027) (Table 1).

**Table 1:** Characteristics of study participants

Variables	Total (n)	Percentage (%)
**Age (years)** mean (SD)	25.8 ± 6.0	
**Age of pregnant women (years)**		
≥ 30	246	24.0
20–29	633	61.6
< 20	148	14.4
**Education level**		
No formal education	650	63.3
Primary	133	13.0
Secondary or higher	244	23.7
**Income**		
Low	342	33.3
Middle	342	33.3
High	343	33.4
**Occupation**		
Not Employed	674	65.6
Employed	353	34.4
**Pregnancy trimester**		
First trimester	440	42.8
Second trimester	452	44.0
Third trimester	135	13.2
**Parity**		
More than one	540	52.6
One	487	47.4
**Residential area**		
Semi urban	590	57.5
Rural	437	42.5
**Age at first pregnancy (years)**		
13–19	732	71.8
20–36	288	28.2

The overall prevalence of HBP was 1.4% (14/1027; 95% CI 0.7–2.3). Prevalence rates for each group were as follows: in the group aged 20–29 years (1.4%; 95% CI 0.7–2.7), without formal education (1.7%; 95%CI 0.9–3.0), in low income households (1.7%; 95% CI 0.8–3.9), without occupation (1.5%; 95% CI 0.8–2.8), in the third trimester (3.0%; 95% CI 1.1–7.7), primipara (1.6%; 95% CI 0.8–3.2), and living in rural areas(1.6%; 95% CI 0.8–3.3). These differences were, however, not statistically significant (Table 2).

**Table 2:** Prevalence of high blood pressure among pregnant women

Variables	Total (n)	Prevalence (%)	95% CI^a^
**Age of women (years)**			
≥ 30	246	1.2	0.4–3.8
20–29	633	1.4	0.7 -2.7
< 20	148	1.3	0.3 -5.3
**Education level**			
No formal education	650	1.7	0.9–3.0
Primary	133	0.0	
Seconday and higher	241	1.2	0.4–3.7
**Income**			
Low	342	1.7	0.8–3.9
Middle	342	1.5	0.6- 3.5
High	343	0.9	0.3–2.7
**Occupation**			
Not employed	674	1.5	0.8–2.8
Employed	353	1.1	0.4–3.0
**Pregnancy trimester**			
First trimester	440	0.7	0.2–2.1
Second trimester	452	1.5	0.7- 3.2
Third trimester	135	3.0	1.1–7.7
**Parity**			
More than one	540	1.1	0.5–2.4
One	487	1.6	0.8–3.2
**Residential area**			
Semi-Urban	590	1.2	0.6- 2.5
Rural	437	1.6	0.8–3.3
**Age at first pregnancy (years**)			
13–19	732	1.5	0.8–2.7
20–36	288	1.0	0.3–3.2

^a^CI, Confidence interval

In the multivariable analysis, pregnancy trimester, maternal age, household income, occupation, parity, and residential area were not associated with HBP during pregnancy (Table 3).

**Table 3:** Logistic regression result of High blood pressure and associated factors among pregnant women

Variables		Crude		Adjusted	
	Total (n)	cOR*	95% CI	aOR**	95% CI
**Age of women(years)**					
20–29	633	1		1	
< 20	246	0.94	0.27–3.25	1.13	0.30–4.30
≥ 30	148	1.12	0.27–4.57	0.82	0.18–3.66
**Education level**					
No formal education	650	1			
Primary	133	0.21	0.12–3.56		
Secondary or higher	244	0.81	0.24–2.69		
**Income**					
Low	342	1		1	
Middle	342	0.84	0.27–2.65	0.79	0.25–2.52
High	343	0.53	0.14–1.97	0.51	0.14–1.93
**Occupation**					
Employed	674	1		1	
Not employed	353	0.81	0.27–2.48	0.96	0.29–3.11
**Pregnancy trimester**					
Second trimester	452	1		1	
First trimester	440	0.47	0.13–1.70	0.42	0.11–1.51
Third trimester	135	2.03	0.62–6.65	2.24	0.68–7.40
**Parity**					
More than one	540	1		1	
One	487	1.46	0.52–4.07	1.79	0.54–6.03
**Residential area**					
Semi-urban	590	1		1	
Rural	437	1.35	0.49–3.76	1.91	0.61–5.93

*cOR* crude odds ratio, *aOR* adjusted odds ratio

## Discussion

The prevalence of HBP in pregnancy was low with 1.4% in Burkina Faso, similar to that reported in African (1.5%) and global studies (0.2%–9.2%) [20, 21]. In 2020, Gemechu et al. reported a prevalence of 0.9% (95% CI: 0.4–1.8) for only gestational hypertension in a systematic review and meta-analysis of 70 studies [4]. The prevalence found in our study is relatively high compared to the prevalence of 0.3% for women of childbearing age reported by Jiang et al. in 2022 [12]. However, large differences have been reported in the prevalence of HBP [8, 20, 20, 22–24]. Berhe et al., reported a range of 8–46.4% in the prevalence of HBP in a systematic review of 258,602 pregnant women [25]. These differences could be explained by the participants’ age. A survey of HBP in China showed a prevalence of 12.7% in pregnant women aged ≥ 40 years compared to a prevalence of 4.3% in pregnant women aged 25–29 years [26]. HBP measurement methods have not been described in all studies therefore different methods may partially explain the differences in prevalence [4, 25]. In addition, differences might be due to sociocultural variability in maternal risk factors and differences in antenatal care accessibility [4].

Most prevalence studies, however, were conducted in referral hospitals and in urban settings where women with hypertension are concentrated for better management [7, 8, 11, 20, 23]. In Togo, the prevalence of hypertension was 12.5% in in a maternity department in an urban area [22]. In Burkina Faso, in the teaching hospital of an urban area, a prevalence of 9.6% was reported [18]. This is similar to the prevalence of 9.9% reported in a health facility in Ethiopia and 9.7% in a Nigerian teaching hospital [24, 25]. Our study was conducted at a first-level health facility. In our sample, more than 76% of the participants were < 30 years of age. Their young age and rural residential environment could explain the observed low prevalence. HBP may be more common in women aged > 35 years, although a high prevalence in women < 30 years of age has been reported in China and Ethiopia [25, 26].

Higher prevalence of hypertension among women in the third trimester of pregnancy (3%) than in the first trimester (0.7%) can be explained by the development of hypertensive disorders of pregnancy after 20 weeks [7]. Differences in prevalence rates in the literature also depend on the stage of pregnancy blood pressure measurement was done, considering the mid-pregnancy blood pressure drop occurring in uncomplicated pregnancies and the level of screening before pregnancy in different countries [27–29].

Maternal age, household income, occupation, parity, and residential area were not associated with HBP during pregnancy in our study. Several studies have reported an association between sociodemographic characteristics [12, 20, 25]. This difference may be explained by the lack of statistical power because we conducted this study to identify the prevalence of HBP during pregnancy [30].

### Study strengths and limitations

This study is the first to assess the prevalence of HBP in the first-level health care system in Burkina Faso. The measurements were performed in a private room after the ANC visit. We took three blood pressure measurements and the average of the last two measurements was considered as the woman's blood pressure. Postpartum pre-eclampsia could not be observed owing to the design of our study. The study lacked statistical power to show statistically significant effects.

## Conclusion

The prevalence of HBP among pregnancy women in the first level of health system care is significantly lower compared to prevalence’s reported in hospital studies. Pregnant women in resource-limited countries face a double burden due to maternal infections and common pathologies such as the hypertensive disorders that compromise perinatal and maternal outcomes of pregnancy. Public health surveillance, primary prevention activities, early screening, and treatment of HDP should be reinforced in all health facilities to reduce the burden of adverse pregnancy outcomes in Burkina Faso.

## Data Availability

Data sets are available from the corresponding author.

## Abbreviations

ANC: Antenatal Care
HBP: High Blood Pressure
HDP: Hypertensive Disorders of Pregnancy
CI: Confidence Interval
aOR: Adjusted Odds Ratio
cOR: Crude Odds Ratio
mmHg: Millimeter of mercury
WHO: World Health Organization
